# Prevalence of abnormalities in electrocardiogram conduction in dialysis patients: a comparative study

**DOI:** 10.1590/2175-8239-JBN-2020-0018

**Published:** 2020-07-20

**Authors:** Firas Ajam, Arda Akoluk, Anas Alrefaee, Natasha Campbell, Avais Masud, Sushil Mehandru, Mayukumar Patel, Arif Asif, Michael P. Carson

**Affiliations:** 1 Jersey Shore University Medical Center - Hackensack Meridian Health, Department of Medicine, Neptune, NJ, EUA.

**Keywords:** Renal Insufficiency, Chronic, Renal Dialysis, Electrocardiography, Insuficiência Renal Crônica, Diálise Renal, Eletrocardiografia

## Abstract

**Background:**

The electrocardiogram (ECG) can aid in identification of chronic kidney disease (CKD) patients at high risk for cardiovascular diseases. Cohort studies describe ECG abnormalities in patients on hemodialysis (HD), but we did not find data comparing ECG abnormalities among patients with normal kidney function or peritoneal dialysis (PD) to those on hemodialysis. We hypothesized that ECG conduction abnormalities would be more common, and cardiac conduction interval times longer, among patients on hemodialysis vs. those on peritoneal dialysis and CKD 1 or 2.

**Methods:**

Retrospective review of adult inpatients’ charts, comparing those with billing codes for “Hemodialysis” vs. inpatients without those charges, and an outpatient peritoneal dialysis cohort. Patients with CKD 3 or 4 were excluded.

**Results:**

One hundred and sixty-seven charts were reviewed. ECG conduction intervals were consistently and statistically longer among hemodialysis patients (n=88) vs. peritoneal dialysis (n=22) and CKD stage 1 and 2 (n=57): PR (175±35 vs 160±44 vs 157±22 msec) (p=0.009), QRS (115±32 vs. 111±31 vs 91±18 msec) (p=0.001), QT (411±71 vs. 403±46 vs 374±55 msec) (p=0.006), QTc (487±49 vs. 464±38 vs 452±52 msec) (p=0.0001). The only significantly different conduction abnormality was prevalence of left bundle branch block: 13.6% among HD patients, 5% in PD, and 2% in CKD 1 and 2 (p=0.03).

**Conclusion:**

To our knowledge, this is the first study to report that ECG conduction intervals are significantly longer as one progresses from CKD Stage 1 and 2, to PD, to HD. These and other data support the need for future research to utilize ECG conduction times to identify dialysis patients who could potentially benefit from proactive cardiac evaluations and risk reduction.

## RESUMO

**Introdução:**

O eletrocardiograma (ECG) pode auxiliar na identificação de pacientes com doença renal crônica (DRC) e alto risco para doenças cardiovasculares. Estudos de coorte descrevem anormalidades no ECG de pacientes em hemodiálise (HD), mas não encontramos dados comparando anormalidades no ECG entre pacientes com função renal normal ou aqueles em diálise peritoneal (DP), com aqueles em hemodiálise. Nossa hipótese foi de que as anormalidades de condução no ECG seriam mais comuns, e o intervalo de condução cardíaca seria mais longo entre os pacientes em hemodiálise comparados àqueles em diálise peritoneal e DRC 1 ou 2.

**Métodos:**

revisão retrospectiva dos prontuários de pacientes adultos internados, comparando aqueles com códigos de cobrança para "Hemodiálise" versus pacientes internados sem esses encargos, e uma coorte de pacientes em diálise peritoneal ambulatorial. Pacientes com DRC 3 ou 4 foram excluídos.

**Resultados:**

Cento e sessenta e sete prontuários foram revisados. Os intervalos de condução no ECG foram consistente- e estatisticamente mais longos entre os pacientes em hemodiálise (n = 88) vs. em diálise peritoneal (n = 22) e DRC estágios 1 e 2 (n = 57): PR (175 ± 35 vs 160 ± 44 vs 157 ± 22 msec) (p = 0,009); QRS (115 ± 32 vs. 111 ± 31 vs 91 ± 18 ms) (p = 0,001); QT (411 ± 71 vs. 403 ± 46 vs 374 ± 55 ms) (p = 0,006 ), QTc (487 ± 49 vs. 464 ± 38 vs 452 ± 52 ms) (p = 0,0001). A única anormalidade de condução significativamente diferente foi a prevalência de bloqueio do ramo esquerdo: 13,6% nos pacientes em HD, 5% em DP e 2% na DRC 1 e 2 (p = 0,03).

**Conclusão:**

Pelo que sabemos, este é o primeiro estudo a relatar que os intervalos de condução no ECG são significativamente maiores à medida que se progride das DRC Estágios 1 e 2, para DP, e para HD. Esses e outros dados corroboram a necessidade de estudos futuros para utilizar os tempos de condução no ECG para identificar pacientes em diálise que poderiam se beneficiar de avaliações cardíacas proativas e assim redução de risco.

## INTRODUCTION

Cardiovascular disease is the leading cause of mortality in patients with chronic kidney disease (CKD). Among patients with end-stage renal disease (ESRD) undergoing hemodialysis (HD), prolonged QTC was associated with higher mortality, but the TpTe/QT was not 1 ^-^ 3. During HD, the QTC interval may become prolonged, and these changes were associated with lower levels of potassium, calcium, and phosphate^4^. In addition to the traditional risk factors such as coronary disease, diabetes, and left ventricular hypertrophy, patients with ESRD have an increased risk of mortality due to electrolyte disturbances, acid-base balance, and plasma volume shifts that occur during hemodialysis 5.

In addition to other modalities, the electrocardiogram (ECG) remains an important method to identify cardiac abnormalities 6. Prior cohort studies found that HD patients commonly had atrial fibrillation, a need for temporary pacing, and a need for permanent pacemaker implantation 7 ^,^ 8. Given the accelerated rate of coronary artery disease and electrolyte abnormalities in this population, we hypothesized that cardiac conduction abnormalities would be more common among HD patients, however we only identified cohort studies and are not aware of comparative data evaluating this question. 

We tested the hypothesis that the prevalence of ECG conduction abnormalities would be higher and cardiac conduction interval times longer among patients on dialysis when compared to those with CKD 1 and 2. We also postulated that the abnormalities would be more common within a hemodialysis cohort compared to a peritoneal dialysis cohort.

## METHODS

A cross-sectional cohort study was carried out utilizing the billing Data Warehouse at Jersey Shore University Medical Center, a 646-bed general medical teaching hospital in Neptune NJ, USA, to identify inpatients over 18 years of age admitted to the medical service. Patients were classified as having received HD if the admission was associated with a billing code for DIA0106504D (Hemodialysis Recurring) or DIA0106501D (Hemodialysis One Time) and their ECGs were compared to inpatients from the same timeframe who did not have a charge for those codes. This methodology would include patients who required HD due to acute kidney injury (AKI).

The primary outcomes were mean conduction intervals and the prevalence of ECG conduction abnormalities among three groups: normal renal function (CKD 1 and 2), those on peritoneal dialysis (PD), and those on hemodialysis (HD). Secondary outcomes included analysis of intervals based on the presence of any medication that could prolong the QT interval, and individual medications. 

Demographics, comorbidities based on physician documentation in the admission and/or progress notes, medications with the potential to prolong the QT interval, lab values, and ECG findings based on documentation by the interpreting cardiologist were collected. Medications that could prolong the QT interval include ondansetron, prochlorperazine, azithromycin, methadone, ciprofloxacin, levofloxacin, haloperidol, HIV protease inhibitors, sotalol, tricyclic antidepressants, amiodarone, antifungal medications (fluconazole only, none were on amphotericin), beta-adrenergic blocking agents, verapamil, and diltiazem. CKD Stages were defined as (mL/min): 1 (eGFR>90); 2 (60-89), 3 (30-59), 4 (15-29), 4 (<15). The following definitions were used: first-degree atrioventricular block (AVB): PR interval > 200 milliseconds; second-degree AVB: gradual increase of the PR interval until a P wave was lost (Mobitz 1) or a fixed PR interval followed by a dropped P wave (Mobitz 2); third degree heart block: QRS waves conducted at their own rate independently from P waves; bundle branch block: a QRS wave originating from a supraventricular electrical activity with a duration > 120; left bundle branch block (LBBB): tall, broad R wave in lead I and V6 lead, and a QS or rS in the V1 lead; right bundle branch block (RBBB): rsR’ wave or a tall, broad R wave in lead V1, and a wide, slurred S wave in lead I and V5-6 leads; prolonged QTc > 440 milliseconds in males and >460 milliseconds in females. The degree of left ventricular dysfunction was categorized based on ejection fraction results from ECG reports and American Society of Echocardiography guidelines: normal 52-72%, mildly abnormal 41-51%, moderately abnormal 30-40%, and severely abnormal <30% 9. Normal ECG intervals were defined as PR 120-200 ms, QRS 60-100 ms, QTC <440 ms in men and <460 ms in women 10.

### STATISTICAL ANALYSIS

Stata 15 (College Park,TX, USA) was used for analysis. Descriptive statistics are reported, mean values were compared using Student’s t-test for normally distributed data, and Wilcoxon rank sum test for non-parametric data. A p-value <0.05 was considered significant.

## RESULTS

Data was collected from 167 patients. The mean age was 69 +17 years, 44% of HD patients, 36% of PD, and 55% of CKD 1 and 2 patients were female. Table 1 lists the demographics and, not surprisingly, a history of the following were more common among HD patients: coronary artery disease, diabetes mellitus, hypertension, and stroke. Among the entire cohort, the percentage with American Society of Echocardiography left ventricular dysfunction categories were: normal 72 (67%), mild 18 (17%), moderate 10 (9%), and severe 7 (6.5%). Regression analysis found no correlation between LV dysfunction category and PR, QRS, QT, QTC. The prevalence of prolonged QTC among the cohorts was 51% (CKD 1-2), 68% (PD), and 77% (HD) and the increase was significant (p=0.004).

**Table 1:** Demographics of patients included in the study.

	CKD 1-2 (n=57)	PD (n=22)	HD (n=88)	K. Wallis
Male	45%	64%	56%	
Average age	61±19	67±16	72±14	
Coronary Artery Disease	16%	45%	50%	p=0.0001
Chronic Obstructive Pulmonary Disease	14%	13%	23%	p=0.34
Atrial Fibrillation	12%	14%	34%	p=0.058
Hypertension	42%	86%	70%	p=0.002
History of Coronary Arterial Bypass Graft	2%	9%	9%	p=0.73
Heart Failure with Reduced Ejection Fraction	5%	5%	17%	p=0.4
Stroke	5%	4	26%	p=0.11
Diabetes Mellitus	21%	36	57%	p=0.0008

Regarding the primary outcomes, there was no difference in the prevalence of conduction blocks by HD status (Table 2), but Table 3 shows that the PR, QRS, QT, and QTC became progressively and significantly longer as patients progressed from CKD 1-2, to PD, to HD. For the entire cohort, the respective mean QRS duration was longer for those on a QT prolonging medication compared to those not on one of those medications (144+29 ms vs. 104+30 ms, p=0.01), and the same was true for the QTC intervals (487+54 ms vs. 470+46, p=0.043). Because the longer intervals seen among ESRD patients could be confounded by the use of QT prolonging medications, we performed a post hoc regression analysis to look for a synergistic effect of ESRD and QT prolonging medications on the intervals. This analysis was limited as only 32 of the ESRD patients were on a medication that could prolong the QT or QTC, and in this cohort we did not find that the mean QRS and QTC were significantly longer among the 32 ESRD patients on a QT prolonging medication. CHF categories, defined as normal>=55%, mild 45-55%, moderate 35-45%, and severe <35%, were not associated with differences in intervals.

**Table 2:** Cardiac Conduction Abnormalities by Dialysis Status.

	CKD 1-2	PD	HD	P value (chi-square)
	(n=57)	(n=22)	(n=88)	
1sr degree heart block	2%	9%	5%	
RBBB	5%	5%	13.6%	0.17
LBBB	2%	5%	13.6%	0.033
LAHB	0	0	3%	
RAE	0%	0%	2%	
LVH	3%	0	12%	

RBBB: right bundle branch block; LBBB: left bundle branch block; LAHB: left anterior hemiblock; RAE: right atrial enlargement; LVH: left ventricular hypertrophy.

**Table 3:** ECG inervals among dialysis verus non-dialysis patients (ms)

	CKD 1-2 (n=57)	DP (n=22)	HD (n=88)	p value (K. Wallis)
PR	157±22	160±44	175±35	0.009
QRS	91±18	111±31	115±32	0.0001
QT	374±55	403±46	411±71	0.0006
QTc	452±42	464±38	487±49	0.0001

## DISCUSSION

This is the first study, to our knowledge, demonstrating that the mean cardiac conduction intervals become longer with normal renal function, PD, and HD when comparing cross-sectional cohorts of patients treated at one institution. Theoretically, this finding can be explained by the physiological and electrolyte derangements becoming more pronounced as patients progress from normal renal function, to daily PD, then thrice weekly HD. Prior studies have reported the relationship between ESRD and QTC prolongation, but their cohorts differed from ours in that they were dichotomous (CKD as eGFR <60 vs. >60 mL/min), included patients with CKD stages 3 and 4, and did not specifically comment on PD patients. One such study that utilized the Cardiovascular Health Study limited database found that ECG abnormalities were more common among those with their definition of CKD including “long QT interval”. However, they defined “long QT interval” as QT>=110 (where QT interval = QT x [heart rate +100]/656]), did not list the actual intervals, and did not sub-stratify by PD or HD status 11. In contrast, our study had 3 cohorts and defined prolonged QTC using accepted sex-based ranges. 

Another study utilizing the Cardiovascular Health Study found the mean QTC to be similar among those with eGFR <60 (429+22, n=600) vs. those with an eGFR >60 (427+21, n=2638), and the respective rates of QT >450 were 17 and 14% 12. Compared to that study, the mean QTC in our cohorts was longer, and the prevalence of QT prolongation was higher, as it was in another cohort of ESRD patients (41% (n=68)).^13^

Therefore, while other confounders may have been present in our population vs. the historical studies, the data showing that intervals became longer as the cohorts progressed to PD and HD strengthens the findings of our study.

Those papers and other documents report that as QT prolongs in patients with ESRD the mortality rate increases^1^^,^ 11 ^,^ 12 ^,^ 14. Most notably, within a cohort of patients with ESRD waiting for transplant, those with a prolonged QTC (39%) had 1-, 3-, and 5-year death-rates of 12%, 36%, and 47%, respectively, vs 8, 24, and 36% for those with normal QTC (log-rank p=0*.*03) 1.

Patients on dialysis often take supplements such as phosphate-binders and sodium bicarbonate, and are subjected to shifts in hydrogen and potassium ions throughout their dialysis sessions. This could explain why all ECG intervals (PR, QRS, QT and QTC) were longer than in the non-dialysis patient population. However, it is worth noting that the mean PR, QRS, and QT intervals in our cohort, while longer among PD and HD patients, were within normal limits.

Our methodology potentially captured patients who had HD due to AKI, but our rationale was to optimize the number of the patients in the sample, and while AKI patients may not have had chronic metabolic derangements, the need for HD would be associated with enough metabolic derangements that could affect the QTC. Theoretically, if QTC prolongation was only found in patients on long term HD, there would be a bias towards the null (no difference in QTC between the normal, PD, and HD groups), so finding a difference suggests that this potential bias was not significant, and our findings are robust.

The impact of ESRD and HD-associated electrolyte abnormalities and shifts on ECG findings have been studied. It was hypothesized that rapid changes in volume and electrolyte concentrations in dialysis patients would result in ECG changes and arrhythmias despite these patients having no prior cardiac history. However, no effect on QRS interval axis before and after HD in patients with ESRD was observed 15. One study did find a correlation between ultrafiltration volumes and the length of the QTC interval 16. Lastly, a prospective trial of 141 HD patients found that the QTC interval was more likely to become longer at the peak of the HD session among those with higher levels of calcium and phosphate and lower levels of potassium and brain natriuretic peptide 4.

While diastolic dysfunction is more prevalent in those with hypertension, CKD, and ESRD, the largest cohort (n=3,487) found that progressive renal dysfunction was associated with a higher prevalence of left ventricular hypertrophy and abnormal LV geometry, but the authors did not detect significant associations between kidney function and systolic or diastolic function after adjusting for potential confounding variables 17. Two studies of 153 and 129 patients found that diastolic dysfunction >grade 1 had a 3.42 (95%CI [1.7,7.1]) HR (hazard ratio) of mortality, and advanced diastolic dysfunction was predictive of cardiovascular events (HR 2.2; 95%CI [1.1,4.3]).^(18, 19)^. 

There is no doubt that ESRD patients are at higher risk for death from cardiovascular causes, and our finding that patients on dialysis were more likely to have prolonged QTC intervals suggests that ventricular arrhythmias could play a role, thus it is important to identify comorbid conditions 11. Patients with CKD have 1,25-dihydroxyvitamin D deficiency due to increased production of fibroblast growth factor 23 (FGF23) and some also have a 25hydroxyD deficiency due to loss of the 1-alpha-hydroxylase enzyme from structural renal compromise, and these deficiencies predispose to hypocalcemia, which could explain the higher prevalence of a prolonged QTC^20^^,^ 21.

As opposed to prior studies that simply reported the prevalence of ECG abnormalities in single cohorts of ESRD patients or compared dichotomous cohorts with eGFR <60 vs. >60, our study is the first to compare cohorts along a spectrum of renal disease from none, to PD, to HD. We found a study that did not find QTC prolongation among PD patients, but unlike other studies, the authors also did not find a correlation between electrolyte abnormalities and ECG intervals 13. Our sample size was similar to prior historical studies.

Potential limitations of this study include that it is from a single center and the prevalence of QTC prolongation was higher than prior reports. This could be related to our studying an inpatient population who are more likely to be acutely ill and have associated electrolyte abnormalities, and this is supported by the prevalence of prolonged QTC intervals in our CKD 1 and 2 population being higher than prior reports. Our primary goal was to report the prevalence of ECG abnormalities in the 3 groups, and the study was not designed to run a multivariable model. This limitation could be addressed in a future study with a sample size large enough to control for multiple other comorbidities. 

Our principal findings were that as patients progress to settings with higher a probability of electrolyte abnormalities and shifts, their ECG intervals become significantly longer. Cardiovascular events and death are higher among those with ESRD and on HD, and our data adds evidence to the literature that ventricular arrhythmias could be a significant cause. While some might apply a logical approach to obtain non-invasive cardiac testing in asymptomatic PD/HD patients with prolonged ECG intervals, we are not aware of data showing that utilization of those healthcare resources will actually improve patient outcomes. Therefore, our ultimate hope is that future research will utilize this ECG data to identify high-risk patients on PD and HD, formally determine if testing/interventions actually improve patient outcomes, and develop clinically effective interventions to reduce their risk of cardiovascular morbidity and death. 
